# Supplementary figures and images for: Assessing the vulnerability of marine life to climate change in the Pacific Islands region
Source: PLoS One. 2022 Jul 8;17(7):e0270930. doi: 10.1371/journal.pone.0270930 (PMC9269963; doi:10.1371/journal.pone.0270930)

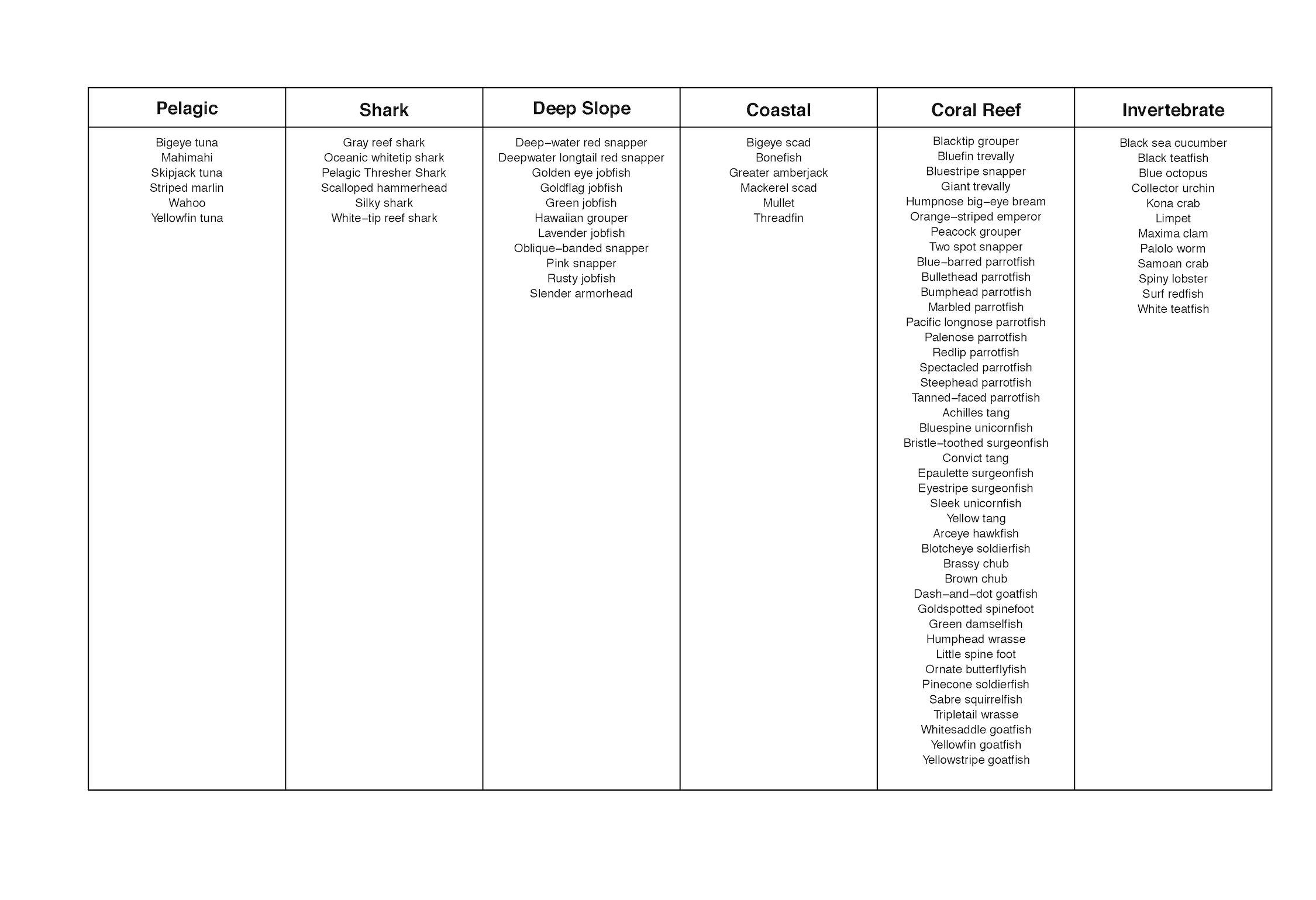

Supplement: S1 Table — (TIFF) [file pone.0270930.s001.tiff]

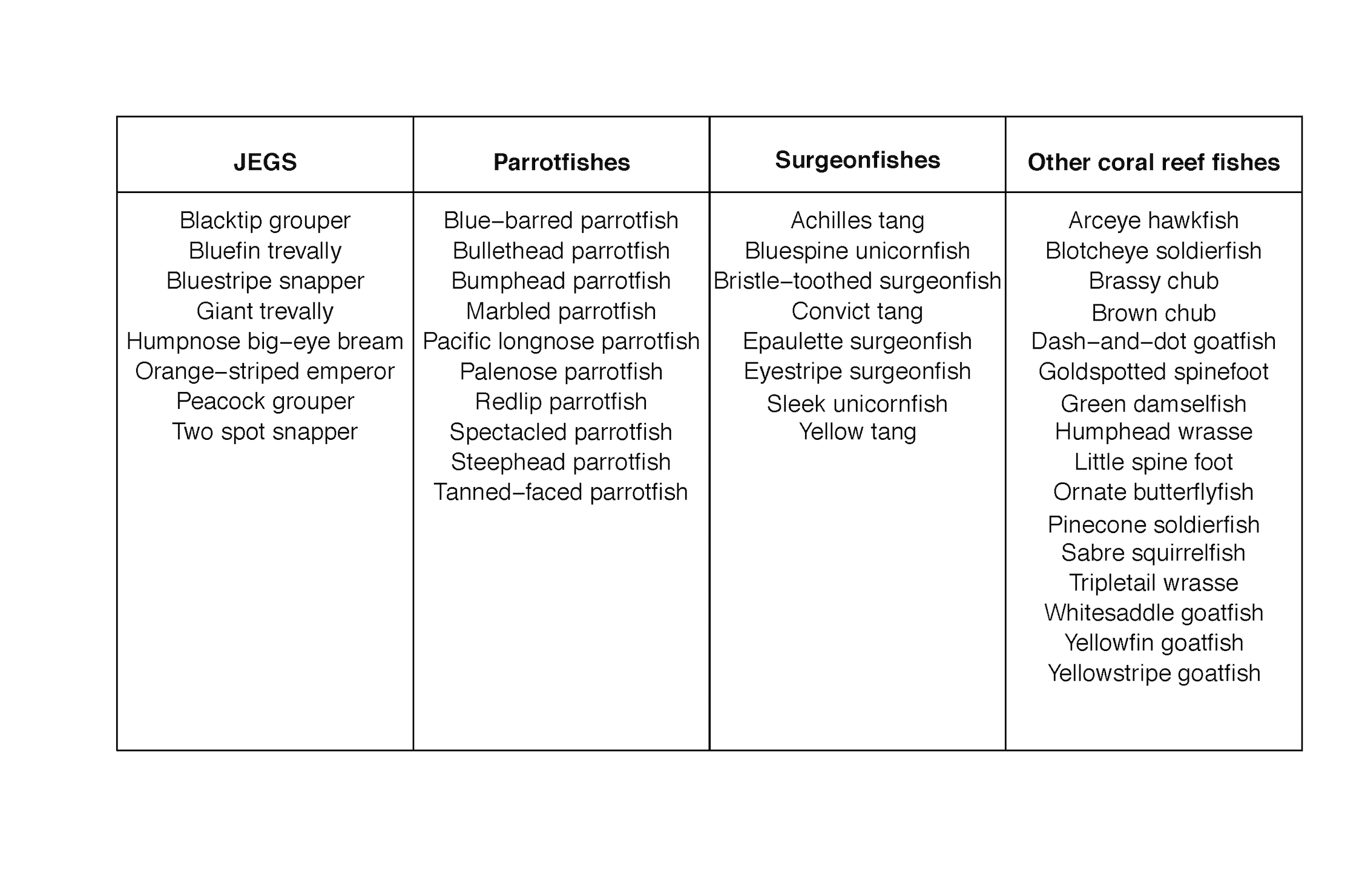

Supplement: S2 Table — (TIFF) [file pone.0270930.s002.tiff]

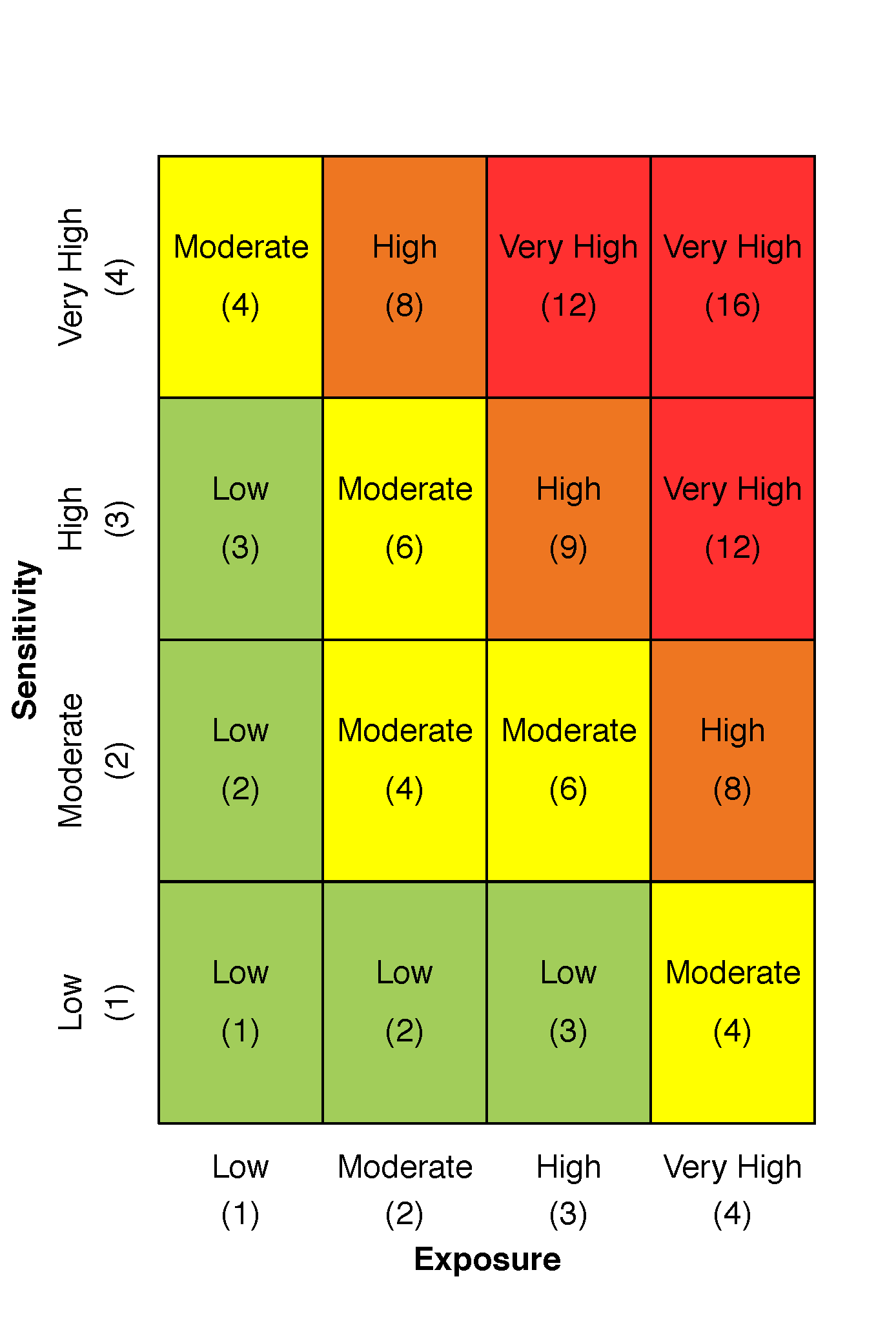

Supplement: S1 Fig — The possible range of these scores is between 1 and 16. The numerical values for the climate vulnerability rank are as follows: 1–3 Low (green), 4–6 Moderate (yellow), 8–9 High (orange), and 12–16 Very High (red). (TIF) [file pone.0270930.s006.tif]
