# Supplementary material for: Assessing the vulnerability of marine life to climate change in the Pacific Islands region
Source: PLoS One. 2022 Jul 8;17(7):e0270930. doi: 10.1371/journal.pone.0270930 (PMC9269963; doi:10.1371/journal.pone.0270930)
Supplement: S1 File — (DOCX) [file pone.0270930.s003.docx]

Exposure Supplement

The Pacific Islands Fisheries Science Center conducted a climate change vulnerability assessment for six groups of marine species in the Pacific Islands region. This supplemental information summarizes the exposure portion of the vulnerability assessment.

To characterize the expected climate change in a particular region of the ocean in a method consistent with established RVA protocol, grids of standardized anomalies for all exposure variables were downloaded and subsetted to match the current distribution of each species. Species distribution maps were obtained from online sources (OBIS; fishbase.net; sealifebase.net) and gridded for use as masks to subset the exposure variable grids. Global grids of modeled exposure variable standardized anomalies were obtained from the NOAA Earth System Research Laboratory (ESRL) Climate Change Web Portal at https://[www.esrl.noaa.gov/psd/ipcc/ocn/.](http://www.esrl.noaa.gov/psd/ipcc/ocn/) Averages of all available model runs (n=23) were used for each variable under consideration using the climate change forecast scenario of RCP8.5.

Data were represented in 0.5-degree latitude and longitude resolution grids of standardized anomalies of future climate change. This standardized anomaly represents the difference in the mean climate in the future time period (2006–2055) compared to the historical reference period (1956–2005), standardized by the de-trended interannual standard deviation for the historical reference period (1956–2005). Variables downloaded from the ESRL data portal included: Mixed Layer Depth, Bottom Salinity, Bottom Temperature, Chlorophyll, Productivity, Sea Surface Temperature, Salinity, Surface Oxygen, Surface pH, Precipitation, Windstress EW, Windstress NS, and Windstress Magnitude.

Exposure scoring was accomplished by defining cutoff values of the standardized anomaly for each pixel across the species distribution. Positive and negative anomalies were treated separately by keeping signage intact. For positive anomalies, the pixel values were tabulated into exposure bins defined by values greater than 0 and less than or equal to 0.5 for Low (1), greater than 0.5 and less than or equal to 1.5 for Moderate (2), greater than 1.5 and less than or equal to 2 for High (3), and greater than 2 for Very High (4). Negative anomaly values were treated similarly using identical cutoffs that were negatively signed.

Histograms of pixel scores were generated for positive and negative standardized anomalies for qualitative visualization purposes. For the final exposure calculation for a given variable and species, positive and negative pixel scores (Low = 1, Moderate = 2, etc.) were aggregated and averaged.

Table of cutoffs

| **Standardized Anomaly Signage** | **Pixel Value** | **Pixel Exposure Score** |
| --- | --- | --- |
| + | 0 < pixel ≤ 0.5 | 1 (Low) |
| + | 0.5 < pixel ≤ 1.5 | 2 (Moderate) |

| + | 1.5 < pixel ≤ 2.0 | 3 (High) |
| --- | --- | --- |
| + | 2.0 < pixel | 4 (Very High) |
| - | -0.5 ≤ pixel < 0 | 1 (Low) |
| - | -1.5 ≤ pixel < -0.5 | 2 (Moderate) |
| - | -2.0 ≤ pixel < -1.5 | 3 (High) |
| - | pixel < -2.0 | 4 (Very High) |

Pacific-wide exposure and spatial subsets of each exposure variable and taxon were created to better characterize the regional variability in exposure for the wide-ranging PIVA taxa. Four spatial subsets were created to examine exposure in the Hawaiian Archipelago, American Samoa, Mariana Islands Archipelago (including Guam and the Commonwealth of the Northern Mariana Islands), and a portion of the Pacific Basin that encompassed the U.S. Pacific Remote Island Areas (PRIAs) of Baker Island, Howland Island, Jarvis Island, Johnston Atoll, Kingman Reef, Palmyra Atoll, and Wake Island. Individual summary visualizations were created for each of the spatial domain, taxon, and exposure variable combinations. Each summary included a pair of histograms for exposure tabulations with respect to signage of projected perturbations relative to baseline (negative for decreases, positive for increases) and overall exposure combining positive and negative perturbations. This absolute value of exposure scores was averaged and included on the visualization.

The following shell script code accesses the standardized anomaly netcdf files, masks for the habitat and spatial subsets, and then generates composite visualizations of exposure maps with summary bar charts using the mapping software Generic Mapping Tools (GMT version 6.0). See <https://www.generic-mapping-tools.org/>for more information about GMT.

# Shell Script Code



#!/bin/ksh

# modified for MacBook with GMT6, does PIVA exposure plots from netcdf # matches to environmental field

# makes maps of std anomaly and a 2 histograms above map # adds thumbnail image

psfile=Exposure.ps

for region in Pacific American_Samoa Hawaii Marianas_Guam PRIAs; do

if [[ $region == "Pacific" ]] then

range=90/300/-60/60 scale=0.056d/0.056d annot=f10a20::/f10a20::WESN xtext=195

ytext=-55 fi

if [[ $region == "American_Samoa" ]] then

range=180/200/-20/-8 scale=0.575d/0.575d annot=f1a2::/f1a2::WESN xtext=190

ytext=-19.5 fi

if [[ $region == "Hawaii" ]] then

range=172/215/11/36 scale=0.273d/0.273d annot=f2a4::/f4a4::WESN xtext=193.5

ytext=12 fi

if [[ $region == "Marianas_Guam" ]] then

range=132/158/10/25 scale=0.45d/0.45d annot=f1a2::/f1a2::WESN xtext=145

ytext=10.5 fi

if [[ $region == "PRIAs" ]] then

range=154/208/-5/27 scale=0.215d/0.215d annot=f2a4::/f2a4::WESN xtext=181

ytext=-4 fi

# Fix region labels to avoid underscore and the CNMI edit if [[ $region == "Pacific" ]]

then

regionlabel="Pacific"

fi

if [[ $region == "American_Samoa" ]] then

regionlabel="American Samoa" fi

if [[ $region == "Hawaii" ]] then

regionlabel="Hawaii" fi

if [[ $region == "Marianas_Guam" ]] then

regionlabel="CNMI & Guam" fi

if [[ $region == "PRIAs" ]] then

regionlabel="PRIAs" fi

for grdfile in `ls ~/PIVA/GRD/*.grd` ; do

gmt grdmath $grdfile $region.grd OR = subset2.grd gmt grdclip subset2.grd -SrNaN/0 -Gsubset.grd

gmt grdinfo $region.grd gmt grdinfo subset.grd

for exposure in MixedLayerDepth BottomSalinity BottomTemperature Chlorophyll Productivity SST Salinity SurfaceOxygen Surface_pH Precipitation Windstress_EW Windstress_NS Windstress_Magnitude; do

if [[ $exposure == "MixedLayerDepth" ]] then

exposurelabel="Mixed Layer Depth" fi

if [[ $exposure == "BottomSalinity" ]] then

exposurelabel="Bottom Salinity" fi

if [[ $exposure == "BottomTemperature" ]] then

exposurelabel="Bottom Temperature" fi

if [[ $exposure == "Chlorophyll" ]] then

exposurelabel="Chlorophyll" fi

if [[ $exposure == "Productivity" ]] then

exposurelabel="Productivity" fi

if [[ $exposure == "SST" ]] then

exposurelabel="SST" fi

if [[ $exposure == "Salinity" ]] then

exposurelabel="Surface Salinity" fi

if [[ $exposure == "SurfaceOxygen" ]] then

exposurelabel="Surface Oxygen" fi

if [[ $exposure == "Surface_pH" ]] then

exposurelabel="Surface pH" fi

if [[ $exposure == "Precipitation" ]] then

exposurelabel="Precipitation" fi

if [[ $exposure == "Windstress_EW" ]] then

exposurelabel="Windstress EW" fi

if [[ $exposure == "Windstress_NS" ]] then

exposurelabel="Windstress NS" fi

if [[ $exposure == "Windstress_Magnitude" ]] then

exposurelabel="Windstress Magnitude" fi

jpgfile=$region"_"`basename $region_$grdfile ".grd"`_$exposure.jpg echo Creating $jpgfile

genus_species=`basename $grdfile ".grd"` genus=`echo $genus_species | awk -F_ '{print $1}` species=`echo $genus_species | awk -F_ '{print $2}`

common=`grep $genus_species PIVA_species_names.csv | awk -F, '{print $2}'` infile="~/PIVA/RCP8.5_"$exposure"_Avg_StdAnom.nc?anomaly"

gmt grd2xyz $infile | awk '{if ($3 != "NaN") print }' |\

gmt nearneighbor -GMatchup3.grd -I0.5/0.5 -r -R0/360/-90/90 -N4/2 -S1.5 gmt grdinfo Matchup3.grd

gmt grdclip subset.grd -Sr0/NaN -Ghabitat.grd

gmt grdmath Matchup3.grd habitat.grd OR = keep.grd gmt grdinfo keep.grd

gmt psbasemap -R$range -Jx$scale -K -B$annot > $psfile

gmt grdimage keep.grd -Cstdanomaly.cpt -R -J -O -K >> $psfile

gmt grdcontour subset.grd -CMatchup2.cnt -W1.5,black -R -J -O -K >>

$psfile

gmt psxy eez_all.xy -: -W0.5,black,- -R -J -O -K >> $psfile

gmt grdimage ~/topo62pos.grd -Q -Jx -R -O -K -Chaxby_globe.cpt >> $psfile gmt pscoast -Jx -R -Df -W0.5,black -O -K >> $psfile

# put in label at bottom of map

echo $xtext $ytext $regionlabel " : " $genus $species " : " $common " : "

$exposurelabel |\

gmt pstext -F+a0,+f17p,Helvetica-BoldOblique,black,+jCM -Jx -R -O -K >>

$psfile

# put in the colorscale over the continent if [[ $region == "PRIAs" ]]

then

xc=5.65i yc=6.65i

else

xc=1.65i yc=6.65i

fi

gmt psscale -D$xc/$yc/7.0c/0.71ch -Tgwhite -O -K -Cstdanomaly.cpt

-B1.0:"Standardized Anomaly":/:: >> $psfile

gmt grd2xyz keep.grd | awk '{if ($3 != "NaN") print $3}' > Matchup3.keep ntotal=`awk '{print $1}' Matchup3.keep | wc -l`

bin1=`awk '{if ($1 <= -2) print $1}' Matchup3.keep | wc -l`

bin2=`awk '{if ($1 <= -1.5 && $1 > -2) print $1}' Matchup3.keep | wc -l` bin3=`awk '{if ($1 <= -0.5 && $1 > -1.5) print $1}' Matchup3.keep | wc -l` bin4=`awk '{if ($1 <= 0 && $1 > -0.5) print $1}' Matchup3.keep | wc -l` bin5=`awk '{if ($1 > 0 && $1 <= 0.5) print $1}' Matchup3.keep | wc -l` bin6=`awk '{if ($1 > 0.5 && $1 <= 1.5) print $1}' Matchup3.keep | wc -l` bin7=`awk '{if ($1 > 1.5 && $1 <= 2) print $1}' Matchup3.keep | wc -l` bin8=`awk '{if ($1 > 2) print $1}' Matchup3.keep | wc -l`

bin18=`echo "scale=2; $bin1+$bin8" | bc` bin27=`echo "scale=2; $bin2+$bin7" | bc` bin36=`echo "scale=2; $bin3+$bin6" | bc` bin45=`echo "scale=2; $bin4+$bin5" | bc`

pbin1=`echo "scale=2; 100*$bin1/$ntotal" | bc` pbin2=`echo "scale=2; 100*$bin2/$ntotal" | bc` pbin3=`echo "scale=2; 100*$bin3/$ntotal" | bc` pbin4=`echo "scale=2; 100*$bin4/$ntotal" | bc` pbin5=`echo "scale=2; 100*$bin5/$ntotal" | bc` pbin6=`echo "scale=2; 100*$bin6/$ntotal" | bc` pbin7=`echo "scale=2; 100*$bin7/$ntotal" | bc` pbin8=`echo "scale=2; 100*$bin8/$ntotal" | bc` pbin18=`echo "scale=2; 100*$bin18/$ntotal" | bc` pbin27=`echo "scale=2; 100*$bin27/$ntotal" | bc` pbin36=`echo "scale=2; 100*$bin36/$ntotal" | bc` pbin45=`echo "scale=2; 100*$bin45/$ntotal" | bc`

echo $bin1 $pbin1 echo $bin2 $pbin2 echo $bin3 $pbin3 echo $bin4 $pbin4 echo $bin5 $pbin5 echo $bin6 $pbin6 echo $bin7 $pbin7 echo $bin8 $pbin8 echo $bin18 $pbin18 echo $bin27 $pbin27 echo $bin36 $pbin36 echo $bin45 $pbin45

echo 1 $pbin1 Very High > Matchup3.table echo 2 $pbin2 High >> Matchup3.table

echo 3 $pbin3 Moderate >> Matchup3.table echo 4 $pbin4 Low >> Matchup3.table

echo 5 $pbin5 Low >> Matchup3.table

echo 6 $pbin6 Moderate >> Matchup3.table echo 7 $pbin7 High >> Matchup3.table

echo 8 $pbin8 Very High >> Matchup3.table

echo 1 $pbin45 Low > Matchup3a.table

echo 2 $pbin36 Moderate >> Matchup3a.table echo 3 $pbin27 High >> Matchup3a.table

echo 4 $pbin18 Very High >> Matchup3a.table

max=`sort -nrk2,2 Matchup3.table | awk '{print $2}' | head -1 ` axismax=`echo "scale=0; (($max/10)+2)*10" | bc`

offset=`echo "scale=2; ($max/15)" | bc` echo $max $axismax $offset

max2=`sort -nrk2,2 Matchup3a.table | awk '{print $2}' | head -1 ` axismax2=`echo "scale=0; (($max2/10)+2)*10" | bc`

offset2=`echo "scale=2; ($max2/15)" | bc` echo $max2 $axismax2 $offset2

echo 4.5 0 > dashed.txt

echo 4.5 $axismax >> dashed.txt

exposureavg=`echo "scale=2; (($pbin45*1)+($pbin36*2)+($pbin27*3)+($pbin18*4))/100 " | bc` echo Exposure average is $exposureavg

gmt psbasemap -R0/9/0/$axismax -JX5.6/4 -O -K -Bf1::/f10a10:Percent:WeSn

-X0 -Y8 >> $psfile

awk '{print $1, $2, $1, 0.55}' Matchup3.table |\

gmt psxy -Sb -JX -O -K -R -CMatchup2a.cpt >> $psfile

awk '{print $1, $2+OFFSET, $2}' OFFSET=$offset Matchup3.table |\

gmt pstext -N -F+a0,+f15p,Helvetica-BoldOblique,black,+jCM -JX -R -O -K >>

$psfile

labelpos1=`echo "scale=2; -1*($axismax/20)" | bc` labelpos2=`echo "scale=2; -1*($axismax/8)" | bc` echo $labelpos1 $labelpos2

awk '{print $1, LP, $3, $4}' LP=$labelpos1 Matchup3.table |\

gmt pstext -N -F+a0,+f12p,Helvetica-Bold,black,+jCM -JX -R -O -K >>

$psfile

echo 2.5 $labelpos2 Negative |\

gmt pstext -N -F+a0,+f22p,Helvetica-Bold,black,+jCM -JX -R -O -K >>

$psfile

echo 6.5 $labelpos2 Positive |\

gmt pstext -N -F+a0,+f22p,Helvetica-Bold,black,+jCM -JX -R -O -K >>

$psfile

#add a dashed line separating out the negative and positive sectors of this left histogram

gmt psxy dashed.txt -W1,black,dashed -O -K -R -JX >> $psfile

gmt psbasemap -R0/9/0/$axismax -JX5.6/4 -O -K -Bf1::/f10a10:Percent:WeSn

-X0 -Y0 >> $psfile

# Start the second histogram to right

gmt psbasemap -R0/5/0/$axismax2 -JX5.6/4 -O -K -Bf1::/f10a10:Percent:wESn

-X6 -Y0 >> $psfile

awk '{print $1, $2, $1, 1.05}' Matchup3a.table |\ gmt psxy -Sb -JX -O -K -R -CMatchup3a.cpt >> $psfile

awk '{print $1, $2+OFFSET, $2}' OFFSET=$offset2 Matchup3a.table |\

gmt pstext -N -F+a0,+f15p,Helvetica-BoldOblique,black,+jCM -JX -R -O -K >>

$psfile

labelpos1=`echo "scale=0; -1*($axismax2/20)" | bc` labelpos2=`echo "scale=0; -1*($axismax2/8)" | bc` echo $labelpos1 $labelpos2

awk '{print $1, LP, $3, $4}' LP=$labelpos1 Matchup3a.table |\

gmt pstext -N -F+a0,+f15p,Helvetica-Bold,black,+jCM -JX -R -O -K >>

$psfile

echo $exposureavg 0 |\

gmt psxy -Sa0.25 -Gred -Wblack -N -JX -O -R -K >> $psfile

echo 2.5 $labelpos2 Overall |\

gmt pstext -N -F+a0,+f22p,Helvetica-Bold,black,+jCM -JX -R -O -K >>

$psfile

gmt psbasemap -R0/5/0/$axismax2 -JX5.6/4 -O -Bf1::/f10a10:Percent:wESn -X0

-Y0 >> $psfile

# convert the postscript to jpg and display it gmt psconvert $psfile -A -P -FExposure -Tg convert Exposure.png -quality 100 $jpgfile display $jpgfile

mv $jpgfile ./Exposure_Plots

# add thumbnail pic pngfile="./PICS/THUMBS/"$genus_species".png" echo $pngfile

if [[ -e $pngfile ]] then

if [[ -e ./Exposure_Plots/$jpgfile ]] then

convert $pngfile -resize 40% thumb.png

composite -geometry +300+3300 thumb.png ./Exposure_Plots/$jpgfile composite.jpg

mv composite.jpg ./Exposure_Plots/Composites/$jpgfile #display ./Exposure_Plots/Composites/$jpgfile

fi fi

done done done

Examples of PIVA visualizations from script:


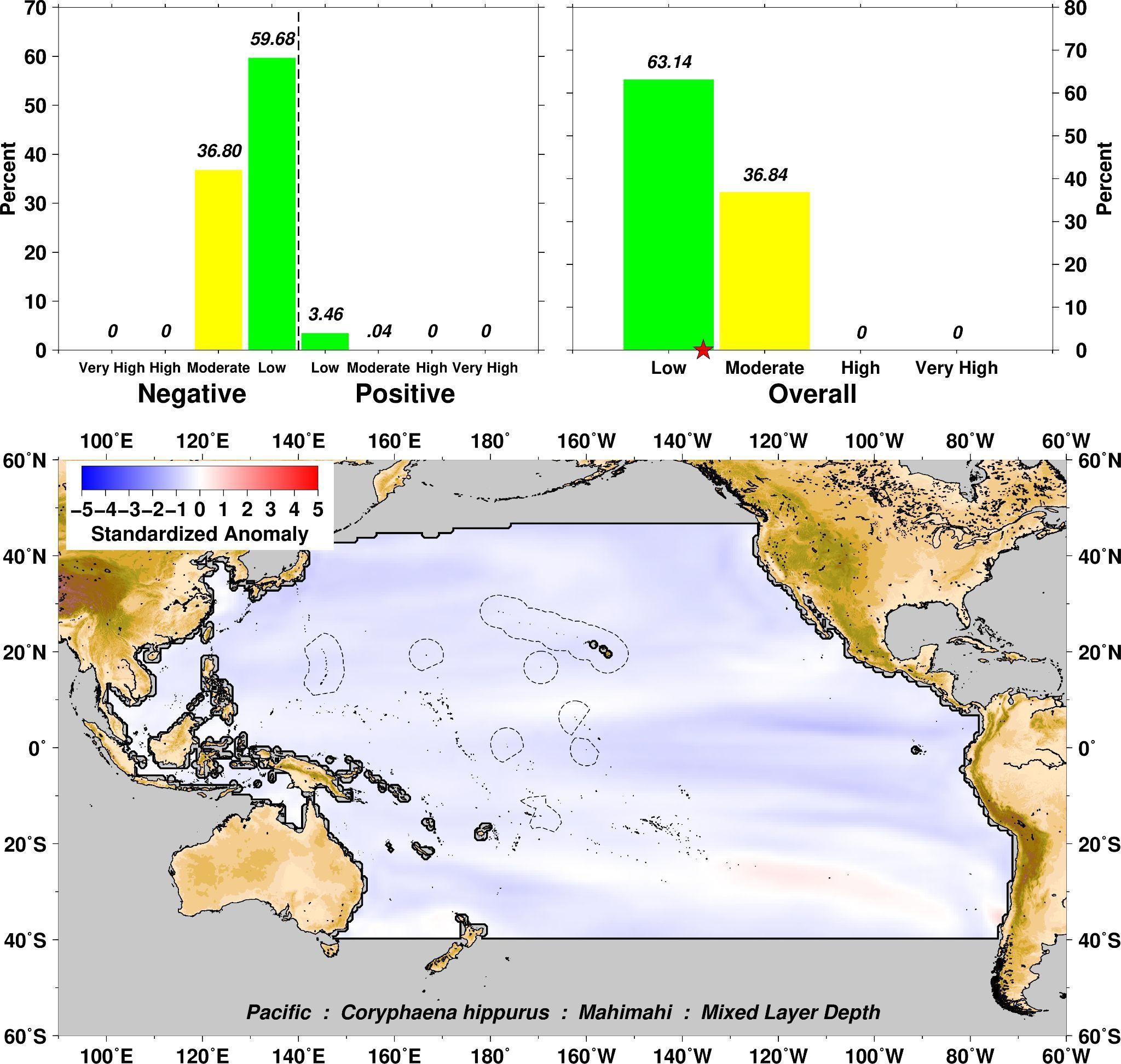


Google Drive folder of all PIVA exposure visualizations: <https://drive.google.com/drive/folders/1J-2Yg6Lljp0aeS27-pMkQgbgmXyjbSgA?usp=sharing>

MPEG-4 video of all PIVA exposure visualizations: <https://drive.google.com/file/d/1u9WgInzNxreL8eU4OwJ8jqgwp8MavwGq/view?usp=sharing>

All map figures in our manuscript and in the supporting information were created by the authors using the free open-source mapping software Generic Mapping Tools (GMT). Additional information for GMT can be found at:

<https://www.generic-mapping-tools.org/>

The GMT visualizations incorporated a public domain database on bathymetry/topography called ETOPO5 which was color-shaded with a GMT color palette called the Haxby color cpt palette file. Additional information on ETOPO5 can be found in:

Data Announcement 88-MGG-02, Digital relief of the Surface of the Earth. NOAA, National Geophysical Data Center, Boulder, Colorado, 1988.

Climate change data used in this study was from the NOAA Climate Change Data Portal. All data located at the portal are publicly provided by the NOAA Physical Sciences Laboratory, Boulder, Colorado, USA. Additional information on this data can be found at:

<https://psl.noaa.gov/ipcc/ocn/>
