# Supplementary material for: Assessing the vulnerability of marine life to climate change in the Pacific Islands region
Source: PLoS One. 2022 Jul 8;17(7):e0270930. doi: 10.1371/journal.pone.0270930 (PMC9269963; doi:10.1371/journal.pone.0270930)
Supplement: S2 File — (PDF) [file pone.0270930.s004.pdf]

Sensitivity \ Exposure	Low	Moderate	High	Very High
Very High				Limpet
High				Arceye hawkfish<br>Black teatfish<br>Collector urchin<br>Maxima clam<br>Oceanic whitetip shark<br>Ornate butterflyfish<br>Pelagic Thresher Shark<br>Scalloped hammerhead<br>Silky shark<br>Surf redfish<br>White teatfish
Moderate				Achilles tang<br>Black sea cucumber<br>Blacktip grouper<br>Bluespine unicornfish<br>Bumphead parrotfish<br>Deep-water red snapper<br>Eyestripe surgeonfish<br>Green damselfish<br>Hawaiian grouper<br>Humphead wrasse<br>Kona crab<br>Little spine foot<br>Palolo worm<br>Slender armorhead<br>Spiny lobster<br>Steephead parrotfish<br>Striped marlin<br>Two spot snapper<br>Whitesaddle goatfish<br>White-tip reef shark<br>Yellow tang
Low				Bigeye scad<br>Bigeye tuna<br>Blotcheye soldierfish<br>Blue octopus<br>Blue-barred parrotfish<br>Bluefin trevally<br>Bluestripe snapper<br>Bonefish<br>Brassy chub<br>Bristle-toothed surgeonfish<br>Brown chub<br>Bullethead parrotfish<br>Convict tang<br>Dash-and-dot goatfish<br>Deepwater longtail red snapper<br>Epaulette surgeonfish<br>Giant trevally<br>Golden eye jobfish<br>Goldflag jobfish<br>Goldspotted spinefoot<br>Gray reef shark<br>Greater amberjack<br>Green jobfish<br>Humpnose big-eye bream<br>Lavender jobfish<br>Mackerel scad<br>Mahimahi<br>Marbled parrotfish<br>Mullet<br>Oblique-banded snapper<br>Orange-striped emperor<br>Pacific longnose parrotfish<br>Palenose parrotfish<br>Peacock grouper<br>Pinecone soldierfish<br>Pink snapper<br>Redlip parrotfish<br>Rusty jobfish<br>Sabre squirrelfish<br>Samoan crab<br>Skipjack tuna<br>Sleek unicornfish<br>Spectacled parrotfish<br>Tanned-faced parrotfish<br>Threadfin<br>Tripletail wrasse<br>Wahoo<br>Yellowfin goatfish<br>Yellowfin tuna<br>Yellowstripe goatfish

Sensitivity (vertical axis); Exposure (horizontal axis)
