# Supplementary material for: Assessing the vulnerability of marine life to climate change in the Pacific Islands region
Source: PLoS One. 2022 Jul 8;17(7):e0270930. doi: 10.1371/journal.pone.0270930 (PMC9269963; doi:10.1371/journal.pone.0270930)
Supplement: S3 File — (PDF) [file pone.0270930.s005.pdf]

Species	Functional Group	Probability of Vulnerability Rank Outcomes			
		Low	Moderate	High	Very High
Black teatfish	Invertebrate	0.00	0.00	0.00	1.00
Collector urchin	Invertebrate	0.00	0.00	0.00	1.00
Limpet	Invertebrate	0.00	0.00	0.00	1.00
Maxima clam	Invertebrate	0.00	0.00	0.00	1.00
Arceye hawkfish	Other Coral Reef	0.00	0.00	0.01	0.99
Ornate butterflyfish	Other Coral Reef	0.00	0.00	0.05	0.96
Oceanic whitetip shark	Shark	0.00	0.00	0.10	0.90
White teatfish	Invertebrate	0.00	0.00	0.12	0.88
Silky shark	Shark	0.00	0.00	0.14	0.86
Scalloped hammerhead	Shark	0.00	0.00	0.14	0.86
Surf redfish	Invertebrate	0.00	0.00	0.26	0.74
Palolo worm	Invertebrate	0.00	0.00	0.37	0.63
Pelagic Thresher Shark	Shark	0.00	0.01	0.42	0.58
Bumphead parrotfish	Coral Reef Parrotfish	0.00	0.00	0.54	0.46
Steephead parrotfish	Coral Reef Parrotfish	0.00	0.00	0.83	0.17
White-tip reef shark	Shark	0.00	0.01	0.89	0.09
Hawaiian grouper	Deep Slope	0.00	0.00	0.92	0.08
Slender armorhead	Deep Slope	0.00	0.00	0.97	0.03
Bluespine unicornfish	Coral Reef Surgeonfish	0.00	0.01	0.96	0.03
Black sea cucumber	Invertebrate	0.00	0.02	0.95	0.03
Eyestripe surgeonfish	Coral Reef Surgeonfish	0.00	0.01	0.97	0.02
Spiny lobster	Invertebrate	0.00	0.02	0.98	0.00
Humphead wrasse	Other Coral Reef	0.00	0.03	0.97	0.00
Green damselfish	Other Coral Reef	0.00	0.02	0.98	0.00
Yellow tang	Coral Reef Surgeonfish	0.00	0.05	0.94	0.01
Kona crab	Invertebrate	0.00	0.06	0.94	0.00
Blacktip grouper	Coral Reef JEGS	0.00	0.09	0.91	0.01
Deep-water red snapper	Deep Slope	0.00	0.16	0.84	0.00

Pink snapper	Deep Slope	0.00	0.23	0.77	0.00	
Whitesaddle goatfish	Other Coral Reef	0.00	0.33	0.66	0.01	
Tanned-faced parrotfish	Coral Reef Parrotfish	0.00	0.38	0.62	0.00	
Sleek unicornfish	Coral Reef Surgeonfish	0.00	0.42	0.58	0.00	
Epaulette surgeonfish	Coral Reef Surgeonfish	0.00	0.43	0.57	0.00	
Striped marlin	Pelagics	0.03	0.38	0.59	0.00	
Bullethead parrotfish	Coral Reef Parrotfish	0.00	0.46	0.54	0.00	
Two spot snapper	Coral Reef JEGS	0.00	0.47	0.53	0.00	
Achilles tang	Coral Reef Surgeonfish	0.00	0.49	0.51	0.00	
Sabre squirrelfish	Other Coral Reef	0.00	0.53	0.47	0.00	
Redlip parrotfish	Coral Reef Parrotfish	0.00	0.61	0.39	0.00	
Spectacled parrotfish	Coral Reef Parrotfish	0.00	0.61	0.39	0.00	
Green jobfish	Deep Slope	0.00	0.64	0.36	0.00	
Gray reef shark	Shark	0.00	0.70	0.30	0.00	
Little spine foot	Other Coral Reef	0.00	0.73	0.27	0.00	
Samoan crab	Invertebrate	0.00	0.74	0.26	0.00	
Blotcheye soldierfish	Other Coral Reef	0.00	0.75	0.25	0.00	
Marbled parrotfish	Coral Reef Parrotfish	0.00	0.80	0.20	0.00	
Peacock grouper	Coral Reef JEGS	0.00	0.82	0.18	0.00	
Humpnose big-eye bream	Coral Reef JEGS	0.00	0.83	0.17	0.00	
Pinecone soldierfish	Other Coral Reef	0.00	0.84	0.16	0.00	
Pacific longnose parrotfish	Coral Reef Parrotfish	0.00	0.85	0.15	0.00	
Threadfin	Coastal	0.00	0.90	0.11	0.00	
Yellowfin goatfish	Other Coral Reef	0.00	0.90	0.11	0.00	
Orange-striped emperor	Coral Reef JEGS	0.00	0.93	0.07	0.00	
Blue octopus	Invertebrate	0.00	0.95	0.06	0.00	
Palenose parrotfish	Coral Reef Parrotfish	0.00	0.95	0.05	0.00	
Goldspotted spinefoot	Other Coral Reef	0.00	0.96	0.04	0.00	
Bonefish	Coastal	0.00	0.96	0.04	0.00	
Dash-and-dot goatfish	Other Coral Reef	0.00	0.98	0.02	0.00	

Rusty jobfish	Deep Slope	0.00	0.98	0.02	0.00	
Deepwater longtail red snapper	Deep Slope	0.00	0.99	0.02	0.00	
Lavender jobfish	Deep Slope	0.00	0.99	0.01	0.00	
Tripletail wrasse	Other Coral Reef	0.00	0.99	0.01	0.00	
Oblique-banded snapper	Deep Slope	0.00	1.00	0.00	0.00	
Convict tang	Coral Reef Surgeonfish	0.00	1.00	0.00	0.00	
Blue-barred parrotfish	Coral Reef Parrotfish	0.00	1.00	0.00	0.00	
Yellowstripe goatfish	Other Coral Reef	0.00	1.00	0.00	0.00	
Bigeye scad	Coastal	0.00	1.00	0.00	0.00	
Bluefin trevally	Coral Reef JEGS	0.00	1.00	0.00	0.00	
Bluestripe snapper	Coral Reef JEGS	0.00	1.00	0.00	0.00	
Brassy chub	Other Coral Reef	0.00	1.00	0.00	0.00	
Bristle-toothed surgeonfish	Coral Reef Surgeonfish	0.00	1.00	0.00	0.00	
Brown chub	Other Coral Reef	0.00	1.00	0.00	0.00	
Giant trevally	Coral Reef JEGS	0.00	1.00	0.00	0.00	
Golden eye jobfish	Deep Slope	0.00	1.00	0.00	0.00	
Goldflag jobfish	Deep Slope	0.00	1.00	0.00	0.00	
Mackerel scad	Coastal	0.00	1.00	0.00	0.00	
Greater amberjack	Coastal	0.00	1.00	0.00	0.00	
Mullet	Coastal	0.01	0.99	0.00	0.00	
Bigeye tuna	Pelagics	0.05	0.96	0.00	0.00	
Mahimahi	Pelagics	0.13	0.87	0.00	0.00	
Skipjack tuna	Pelagics	0.20	0.80	0.00	0.00	
Yellowfin tuna	Pelagics	0.21	0.80	0.00	0.00	
Wahoo	Pelagics	0.22	0.78	0.00	0.00	
