# Supplementary material for: Assessing the vulnerability of marine life to climate change in the Pacific Islands region
Source: PLoS One. 2022 Jul 8;17(7):e0270930. doi: 10.1371/journal.pone.0270930 (PMC9269963; doi:10.1371/journal.pone.0270930)
Supplement: Attachment — Submitted filename: Response to Reviewers_New.docx [file pone.0270930.s007.docx]

**June 1, 2022**

**Good day PLOS One,**

**Please find below our responses in bold to the review of our manuscript. The Editor comments are first, followed by Reviewer 1, Reviewer 2. We have addressed every item that was brought to our attention.**

**Thx. Donald Kobayashi, Gabriella Mukai & the PIVA team**

**Editor**

1. Highlight differences between the study region and other previously assessed regions in the U.S.: Although this ms mostly follows the protocol defined by efforts from previously completed regions, the authors highlight some challenges specific to this region, which is quite different from other U.S. marine ecosystems. These methodological differences could be highlighted in the text.
   - **Changes were made in the introduction and discussion to highlight these challenges and solutions (e.g., quantifying exposure, discussion of hermaphroditism).**
2. Explain the methods more clearly: The methods section can be hard to understand at times, so I urge the authors to address the reviewer comments to address these limitations. In addition to describing some of the methods in more detail, I wonder if you could walk the readers through some specific examples for some taxa that either scored especially high / low, or that proved particularly challenging to assess.
   - **Exposure and sensitivity scoring methodologies were clarified in their respective sections and in the S1 Materials, including enough detail to enable readers to understand vulnerability scoring.**
3. Define exposure scores: It is difficult to determine how the final exposure score for a given attribute, was determined. Based on the text and in the supplement, it seems that it is based on a percentage of grid cells that have different exposure levels. However, it is important to explicitly describe what the different levels mean. For instance, how are the “low” and “medium” levels mean and how are they defined quantitively: how do they relate to the baseline. Please consider adding a table where each of these levels is clearly defined.
   - **Exposure scoring was clarified in the main text and we also provided more details in S1 Materials. A table within S1 Materials provides the cutoff values for each score.**
4. Define the expert scoring system: For the “sensitivity” section, the framework of the expert scoring is unclear? Can you please explain what are the range of possible values that can be assigned for a given factor? Can you also provide an explicit description of what the various individual values mean or represent?
   - **Sensitivity scoring was clarified in the main text. Score values were also provided in the text.**

**Changes to the reference list include: citations for the web portals (#22-28), the NOAA data reports for each species group (#30-35), removal of one citation (#38) since this was incorporated into citations 30-35, an addition of one citation (#80) per the suggestions of a reviewer and another citation (#81) that complements the citation 80.**

**Maps were generated by the authors using Generic Mapping Tools (GMT, free open-source mapping software) and do not fall under copyright restrictions. Topographic data used was ETOPO5 and is in public domain data. Other data layers used in our figures are for environmental variables which are freely available to the public on NOAA Climate Change Data Portal website** [**https://psl.noaa.gov/ipcc/ocn/**](https://psl.noaa.gov/ipcc/ocn/)**.**

**ETOPO5 guidance: "Referencing the Data: The ETOPO5 data may be credited in publications by reference to "Data Announcement 88-MGG-02, Digital relief of the Surface of the Earth. NOAA, National Geophysical Data Center, Boulder, Colorado, 1988"”**

**NOAA Climate Change Data Portal guidance: "Data/image provided by the NOAA Physical Sciences Laboratory, Boulder, Colorado, USA, from their website at https://psl.noaa.gov/ipcc/ocn/".**

**Reviewer 1**

1. Line 281 - is a -1.37% decrease is pH meaningful given that the pH scale is logarithmic?
   - **Originally, we had standardized all the changes/anomalies to percentage. Reviewer 1 brings up an excellent point that describing the change in pH would be more meaningful and clearer if the change was described in pH units rather than percentage. The text has been modified to reflect the changes in pH units and surface oxygen units.**
2. Line 450 - I recommend moving this summary paragraph before the functional groups (at line 356)
   - **This suggestion was incorporated. The paragraph was moved to the suggested place along with the figure. Following figures were relabeled.**
3. Line 462 - The authors describe the high uncertainty for 8 species. A sentence or two on the magnitude of low uncertainty would be useful or a sentence summarizing the number of species with high, moderate, and low uncertainty.
   - **We added a sentence on the breakdown of high and moderate certainty and updated the low certainty species list. We also changed the wording to say “certainty” (instead of “uncertainty”) since we think it’s more intuitive to associate a high number value (i.e., 90%) with high certainty rather than low uncertainty.**
4. Line 598 - Worth mentioning that the analysis is relative.
   - **This suggestion was incorporated and we further clarified the limitations of this analysis.**
5. Given the importance of habitat, it could be useful for the authors to briefly discuss Farr et al (2021) and the potential value of assessing climate change impacts on habitats. <https://journals.plos.org/plosone/article?id=10.1371/journal.pone.0260654>
   - **This suggestion was incorporated in addition to a brief discussion of assessing the impacts on human communities.**
6. Figure 1 could be included in a table.
   - **This suggestion was incorporated. Figure 1 was converted to a table (Table 1). Following tables and figures were relabeled as appropriate.**
7. Figure 3 could be in supplemental materials.
   - **This suggestion was incorporated. Figure 3 was moved to supplementals. Following figures were relabeled as appropriate.**

**Reviewer 2**

1. L85-87: This sentence reads somewhat awkwardly. Recommend reformatting, perhaps starting with something like “The Pacific region offered unique challenges including…….”
   - **This suggestion was incorporated.**
2. L142-143: Here and elsewhere in the manuscript, websites should be more formally described (or at least give the title of the project/database) and also cited. Often in the methods, the sources are only given as a website in parentheses.
   - **This suggestion was incorporated.**
3. L146-148: A little more detail (1-2 sentences) should be added for the climate projection data. Provide some context to “model runs” and give a brief description of what the RCP 8.5 scenario represents.
   - **This suggestion was incorporated. RCP8.5 and “model runs” were defined.**
4. L151-152: Is the historical reference period also from the same climate models? In other words, not from an independent data set. If so, probably worth noting that.
   - **Yes, and we explicitly stated this in the text now.**
5. L187-192: It is still a bit difficult to determine how the final exposure score, for a given attribute, is determined. Based on text here and in the supplement, it seems that it is based on a percentage of grid cells that are low, medium, etc. exposure. I also assume that “low” means within one standard deviation of the baseline, while “medium” is two standard deviations, etc. However, this doesn’t appear to be described anywhere.
   - **Thank you for your comment, this suggestion was incorporated in the main text and we also provided more details in S1 Materials.**
6. L203: For the “Sensitivity” section, not sure if I missed it (sorry if so), but is it stated anywhere what the framework of the expert scoring is? What are the range of possible values that can be assigned for a given factor?
   - **Thank you for your comment, the text has been revised to better explain how experts scored each attribute and the values of each score.**
7. Table-1 heading is missing “Northern”
   - **This suggestion was incorporated.**
8. L212: Change “their” to “them”
   - **This suggestion was incorporated.**
9. L587-588: Was life history complexity treated differently in the other regions where this was done?
   - **Thank you for your comment, we expanded on the challenge of scoring hermaphroditic species and working within the confines of the “Complexity of Reproductive Strategy” attribute. This challenge was not addressed in the original Northeast region RVA.**
